# Job burnout among medical interns: a mixed-methods study of current status, associated factors, and experiential characteristics

**DOI:** 10.3389/fpsyg.2026.1922425

**Published:** 2026-10-07

**Authors:** Zhiyong Sun, Zhao Yin, Zheng Li, Yifan Song, Xiaojing Lu, Kefeng Liu

**Affiliations:** 1 Department of Pharmacy, The First Affiliated Hospital of Zhengzhou University, Zhengzhou, China; 2 Institute for Hospital Management of Henan Province, Zhengzhou, China; 3 Henan Drug Clinical Comprehensive Evaluation Center, Zhengzhou, China

**Keywords:** burnout, clinical internship, emotional exhaustion, medical interns, mixed methods research

## Abstract

**Background:**

Burnout during clinical internships may affect medical students’ learning, professional development, and career choices. We examined the current status, associated factors, and experiential characteristics of burnout among medical interns.

**Methods:**

This study was conducted at a large tertiary hospital in central China. During the quantitative phase, an online cross-sectional questionnaire survey was conducted using the Maslach Burnout Inventory to assess burnout among medical interns and identify factors associated with the total burnout score. During the qualitative phase, semi-structured interviews were conducted to explore the specific manifestations of burnout among medical interns. All interviewees were recruited using purposive sampling; we selected interns whose total burnout scores were at or above the 75th percentile, and the interview data were analyzed using thematic analysis. Finally, the quantitative and qualitative findings were integrated.

**Results:**

A total of 1,054 medical interns participated, and 23 interns were enrolled for semi-structured interviews. The mean total burnout score was 64.40 ± 18.52, while the mean scores for emotional exhaustion, depersonalization, and reduced personal accomplishment were 20.57 ± 9.46, 7.75 ± 5.65, and 36.08 ± 8.71, respectively. All three dimensions were significantly positively correlated with the total burnout score, with emotional exhaustion showing the strongest correlation (*r* = 0.849, *p* < 0.01). Multiple linear regression analysis showed that institutional level, scholarship experience, having a fixed clinical preceptor during the internship, having clearly defined career goals, and experience of medical disputes were significantly associated with the total burnout score. Having clearly defined career goals had the largest standardized regression coefficient (*β* = −0.241, *p* < 0.001). The qualitative findings showed that emotional exhaustion was mainly manifested as physical fatigue, mental depletion, prolonged periods on duty, and insufficient rest. Depersonalization was mainly manifested as a limited understanding of patients, emotional detachment, and indifference toward clinical tasks. Reduced personal accomplishment was mainly manifested as repetitive work, a diminished sense of work-related value, and inadequate opportunities for professional growth.

**Conclusion:**

Burnout among medical interns is distinctly multidimensional, involving physical and mental depletion, emotional detachment, and a diminished sense of professional value. Burnout is associated not only with individual and academic characteristics but also with career planning, preceptor support, and clinical experiences.

## 1 Introduction

Job burnout is a widely recognized occupational health concern in medical education and healthcare (Ratcliff, 2024). The World Health Organization defines burnout as an occupational phenomenon resulting from chronic workplace stress that has not been successfully managed, with core dimensions including emotional exhaustion, depersonalization, and reduced personal accomplishment. Burnout is associated not only with adverse mental health outcomes such as depression, anxiety, fatigue, and sleep problems, but also with reduced quality of care, threats to patient safety, and impaired long-term professional development (Maddock, 2024).

Medical interns are at a critical stage of transition from classroom-based learning to authentic clinical practice and from the role of student to that of a healthcare professional (Jia et al., 2025). Unlike junior medical students, they have entered real-world clinical settings, where they must translate theoretical knowledge into practical competence within a limited period while negotiating their roles among supervisory guidance, patient interaction, departmental responsibilities, and educational assessment (Suh et al., 2023; Cho et al., 2024). Academic demands are further compounded by patient contact, clinical responsibilities, variation in supervision, and career-planning pressure, making their burnout experiences highly contextual and complex (Zhou et al., 2025). Burnout among medical interns should therefore be examined within the broader contexts of the clinical learning environment, role transition, and professional socialization (Di Vincenzo et al., 2024). Previous research suggests that mistreatment, adverse learning experiences, or an unfavorable learning environment are associated with emotional exhaustion, detachment, and career regret (Lu et al., 2023), whereas positive teacher interactions, a supportive emotional climate, and strong peer relationships may be associated with lower burnout (Ren et al., 2024). In China, ongoing healthcare reform, strained physician-patient relationships, medical disputes, and negative public discourse may further heighten medical learners’ concerns about their future working environment (Doleman et al., 2023; Ryan et al., 2023). Early identification of burnout during medical internship and a deeper understanding of its contextual correlates are therefore important for protecting interns’ psychological wellbeing and supporting their professional development.

Previous studies have examined burnout among medical and health professions learners from multiple perspectives (El-Awaisi et al., 2024; Leep Hunderfund et al., 2025; Chen et al., 2024), and our previous research on professional identity formation (PIF) among medical interns also identified burnout as a recurrent issue during clinical internship (Jia et al., 2025). However, burnout levels among medical interns remain substantial, and its specific manifestations and underlying contexts in authentic clinical settings require further clarification. Moreover, most existing studies have relied on cross-sectional questionnaire surveys or secondary data analyses, which are useful for identifying burnout levels and associated factors but are less able to capture how interns experience and interpret burnout in real-world clinical practice (Dall’Ora et al., 2023). There remains a lack of systematic and integrated research examining how emotional exhaustion, depersonalization, and reduced personal accomplishment are manifested through clinical responsibilities, patient contact, supervisory relationships, and experiences of professional growth (Getie et al., 2025). Therefore, this study adopted an explanatory sequential mixed-methods design. First, a cross-sectional questionnaire survey was conducted to describe the current status of burnout among medical interns and identify its associated factors. Subsequently, informed by the quantitative findings, semi-structured interviews were undertaken to explore interns’ experiences of burnout during clinical training. Finally, the quantitative and qualitative findings were integrated to develop a comprehensive understanding of burnout among medical interns, aiming to provide evidence for the early identification of burnout, the optimization of clinical education management, and the development of supportive interventions.

## 2 Methods

### 2.1 Study design and setting

This study was a continuation of our research team’s previous studies (Jia et al., 2025). The study was conducted at a large tertiary Grade A hospital in Zhengzhou, Henan Province, central China, whose basic characteristics have been described previously (Jia et al., 2025). Focusing on the current status, associated factors, and experiential characteristics of burnout among medical interns, this study adopted an explanatory sequential mixed-methods design. In the first phase, a quantitative study was conducted using a cross-sectional questionnaire survey to describe the level of burnout among medical interns and examine its associated factors. In the second phase, a qualitative study was undertaken, in which both participant selection and interview guide development were informed by the quantitative findings, with particular attention given to the burnout-related experiences of interns with relatively high levels of burnout during clinical training. All participants provided written informed consent before taking part in the study. This study was reported in accordance with mixed-methods research reporting guidelines, and the qualitative component was reported with reference to the Consolidated Criteria for Reporting Qualitative Research (COREQ) checklist (Delgado-Galindo et al., 2023), which is presented in Supplementary Table S1 (Supplementary Material).

### 2.2 Participants

#### 2.2.1 Quantitative study participants

A census sampling approach was used in the quantitative phase. Participants were interns from medicine- and health-related programs undertaking clinical training at the study hospital, including students enrolled in four-year bachelor’s degree programs and three-year associate degree programs. The undergraduate students came from institutions of different tiers. Under the current internship management system in China, medical interns with different educational backgrounds generally receive relatively standardized internship management and teaching arrangements after entering the clinical training stage. Students in medicine- and health-related programs typically undertake approximately one year of clinical internship in healthcare institutions during their final academic year, generally beginning in July and ending around April of the following year. The medical interns included in this study were drawn from several medicine-and health-related disciplines, including clinical medicine, rehabilitation therapy, medical laboratory technology, medical imaging technology, and pharmacy.

#### 2.2.2 Qualitative study participants

Purposive sampling was used during the qualitative phase. Participants were recruited from medical interns who had completed the quantitative questionnaire survey. Based on the quantitative findings, only those interns whose total burnout scores were at or above the 75th percentile were included in the qualitative phase and invited to participate in semi-structured interviews. Maximum variation sampling was applied by considering characteristics such as sex, specialty, educational level, and clinical internship experience, with the aim of capturing diverse experiences of clinical training and burnout among medical interns from different backgrounds.

### 2.3 Data collection

#### 2.3.1 Quantitative data collection

Quantitative data were collected using an online questionnaire, which was administered through the Wenjuanxing platform. With the assistance of internship administrators, the researchers distributed the questionnaire to medical interns via QR codes or web links. Participants completed the questionnaire after reading the study information and providing informed consent. The questionnaire consisted of two sections: basic information and the burnout scale. Basic information included sex, age, specialty, the institutional level, scholarship experience, whether the participant had a fixed clinical preceptor during the internship, whether the participant had clearly defined career goals, experience of medical disputes, household income range, and relationship status.

The Maslach Burnout Inventory Human Services Survey (MBI-HSS) used in this study was developed in 1981 by American psychologists Maslach and Jackson (Maslach and Jackson, 1981). The scale consists of 22 items, 9 of which were used for evaluating emotional exhaustion, 5 for evaluating depersonalization, and 8 for evaluating personal accomplishment. Items were assessed on the seven-point Likert scale ranging from 0 (never experienced such a feeling) to 6 (experience such feelings every day), with higher scores indicating a higher level of burnout in the corresponding dimension. As an internationally common scale, the Chinese version of the MBI-HSS has been widely applied in studies (Xie et al., 2021; Sun et al., 2023). The total burnout score was calculated by summing the scores of all items. In the present study, the total burnout score was treated as a continuous variable, and no fixed cut-off values were applied. Before the formal survey, the research team conducted a pilot test to assess the comprehensibility of the questionnaire and the feasibility of its completion.

#### 2.3.2 Qualitative data collection

Qualitative data were collected through semi-structured interviews. The research team developed an interview guide based on the study objectives and the three dimensions of the burnout scale, following an approach used in our previous mixed-methods study on professional identity formation among medical interns (Jia et al., 2025). The preliminary guide was reviewed by a panel of experts and pilot-tested through pilot interviews with eligible interns, after which minor wording revisions were made. The interviews mainly explored medical interns’ overall experiences during clinical training, experiences of physical and psychological exhaustion, experiences of interacting with patients, perceived involvement in clinical tasks, perceptions of the value of their work, and experiences of professional growth. The interviews were conducted by ZL, YFS, XJL, and ZYS, all of whom had received training in qualitative research and had experience in conducting semi-structured interviews. None of the interviewers had a supervisory or evaluative role over the participants. The interviews were conducted by a researcher with experience in qualitative research, while another researcher documented the interview process and prepared field notes. The interviews were generally at the campus where each participant was undertaking their internship, in a quiet, private, and interruption-free setting. Each interview lasted approximately 40 min. With participants’ consent, all interviews were audio-recorded and transcribed verbatim after completion. The transcripts were anonymized before analysis to protect participants’ privacy. Transcripts and preliminary themes were returned to participants for verification, and their feedback was incorporated into the final analysis. Throughout data collection and analysis, the research team continuously assessed the composition of the sample and the richness of the data and adjusted subsequent recruitment based on the preliminary findings. Interviews continued until data saturation was reached, defined as the point at which no new themes, categories, or important information emerged from successive interviews.

### 2.4 Data analysis

#### 2.4.1 Quantitative data analysis

After the online questionnaire data were exported from the Wenjuanxing platform, preliminary data cleaning and organization were performed using Microsoft Excel, followed by statistical analysis using IBM SPSS Statistics version 26.0. Continuous variables were expressed as means and standard deviations, whereas categorical variables were expressed as frequencies and percentages. The total burnout score and the scores for the three dimensions-emotional exhaustion, depersonalization, and reduced personal accomplishment-were calculated based on the scores of the individual scale items. Correlation analysis was performed to examine the relationships among the three dimensions of burnout and the total burnout score. The Shapiro–Wilk test was used to assess the normality of the data. As the burnout scores followed a normal distribution (*p* ≥ 0.05), Pearson’s correlation analysis was employed. Multiple linear regression analysis was conducted to identify factors associated with burnout among medical interns, with the total burnout score as the dependent variable and sex, age, specialty, the institutional level, scholarship experience, whether the intern had a fixed clinical preceptor during the internship, whether the intern had clearly defined career goals, experience of medical disputes, household income range, and relationship status as independent variables. In the multiple linear regression analysis, categorical variables were numerically coded, and multicollinearity was assessed using the variance inflation factor (VIF). All statistical tests were two-sided, and a *p* value of <0.05 was considered statistically significant.

#### 2.4.2 Qualitative data analysis

Qualitative data were analyzed using reflexive thematic analysis, following the six-phase approach described by Braun and Clarke (Braun and Clarke, 2023). The research team first read the interview transcripts repeatedly to become familiar with the data. Initial coding adopted a hybrid deductive–inductive strategy: a deductive coding framework based on the three dimensions of the burnout scale guided initial coding, while inductive codes and subthemes were generated from the participants’ specific accounts in the interview data. Two researchers independently coded the interview transcripts and extracted content related to emotional exhaustion, depersonalization, and reduced personal accomplishment. After coding was completed, the research team discussed the preliminary coding results, compared and revised areas of disagreement, and reached consensus through thorough discussion. The final themes comprised emotional exhaustion, depersonalization, and reduced personal accomplishment. NVivo software was used to manage and analyze the interview data.

### 2.5 Integration of quantitative and qualitative findings

The quantitative and qualitative findings were integrated after the two types of data had been analyzed separately. The integration procedure was guided by established mixed methods integration frameworks (Fetters et al., 2013; O’Cathain et al., 2010). The integration process was organized around the three dimensions of burnout: emotional exhaustion, depersonalization, and reduced personal accomplishment. Following the joint display approach (Guetterman et al., 2015), the research team compared and integrated the quantitative findings for each dimension of burnout with the corresponding experiential characteristics identified in the interview data. A joint display was constructed to present the integrated findings side by side, allowing convergence and divergence between the quantitative and qualitative results to be identified.

## 3 Results

### 3.1 Participant characteristics

A total of 1,194 questionnaires were returned, of which 1,054 were valid (validity rate 88.3%, 1,054/1,194) and were included in this study. Participant characteristics included demographic characteristics, academic background, clinical internship experiences, and factors related to social support, are presented in Table 1. The qualitative study included 23 participants.

**Table 1:** Specific basic information table for study subjects.

Item	Quantitative studies *n* (%)	Qualitative studies *n* (%)
1. Gender		
Male	299 (28.3%)	12 (52.2%)
Female	755 (71.7%)	11 (47.8%)
2. Education level		
First-tier undergraduate	125 (11.8%)	4 (17.4%)
Second-tier undergraduate	365 (34.7%)	7 (30.4%)
College student	564 (53.5%)	12 (52.2%)
3. Scholarship		
Received	386 (36.6%)	6 (26.1%)
Never received	668 (63.4%)	17 (73.9%)
4. Assigned mentor		
Have	642 (60.8%)	14 (60.9%)
None	412 (39.2%)	9 (39.1%)
5. Professional career planning		
Clear	751 (71.1%)	15 (65.2%)
Unclear	303 (28.9%)	8 (34.8%)
6. Experience with medical disputes		
Experienced	91 (8.6%)	1 (4.3%)
Unexperienced	963 (91.4%)	22 (95.7%)
7. Annual household income		
50,000 and below	576 (54.7%)	13 (56.5%)
50,000–100,000	331 (31.4%)	6 (26.1%)
100,000–20,000	110 (10.4%)	3 (13.0%)
200,000–500,000	27 (2.6%)	1 (4.4%)
≥500,000	10 (0.9%)	0 (0.0%)
8. Relationship status		
Has a partner	295 (28.0%)	4 (17.4%)
Without a partner	759 (72.0%)	19 (82.6%)

#### 3.1.1 Burnout levels among medical interns

The burnout scale was used to assess burnout levels among medical interns. The scale comprises three dimensions: emotional exhaustion, depersonalization, and reduced personal accomplishment. The results showed that the mean total burnout score among medical interns was 64.40 ± 18.52. The mean scores for emotional exhaustion, depersonalization, and reduced personal accomplishment were 20.57 ± 9.46, 7.75 ± 5.65, and 36.08 ± 8.71, respectively. The total burnout score and the scores for each dimension are presented in Table 2.

**Table 2:** Total score and subscale scores of job burnout.

Dimension	*N*	Mean	Standard deviation
Emotional exhaustion	1,054	20.5674	9.46301
Depersonalization	1,054	7.7467	5.65092
Reduced personal accomplishment	1,054	36.0816	8.71033
Total job burnout score	1,054	64.3956	18.51859

#### 3.1.2 Correlations among the dimensions of burnout

Correlation analysis was conducted for the total burnout score and its three dimensions. The total burnout score was significantly positively correlated with emotional exhaustion, depersonalization, and reduced personal accomplishment, with correlation coefficients of 0.849, 0.803, and 0.682, respectively (all *p* < 0.01). A strong positive correlation was observed between emotional exhaustion and depersonalization (*r* = 0.723, *p* < 0.01). Emotional exhaustion was significantly positively correlated with reduced personal accomplishment (*r* = 0.251, *p* < 0.01), and depersonalization was also significantly positively correlated with reduced personal accomplishment (*r* = 0.274, *p* < 0.01). Detailed results are presented in Table 3.

**Table 3:** Correlations among dimensions of job burnout.

Dimension	Emotional exhaustion	Depersonalization	Reduced personal accomplishment	Total job burnout score
Emotional exhaustion	1			
Depersonalization	0.723^**^	1		
Reduced personal accomplishment	0.251^**^	0.274^**^	1	
Total job burnout score	0.849^**^	0.803^**^	0.682^**^	1

**p < 0.01.

### 3.2 Multiple linear regression analysis of factors associated with burnout

Multiple linear regression analysis revealed that institutional level, scholarship experience, having a fixed clinical preceptor during internship, having clearly defined career goals, and experience of medical disputes were significantly associated with the total burnout score. Among these factors, having clearly defined career goals (*β* = −0.241, *p* < 0.001), scholarship experience (*β* = −0.062, *p* = 0.009), and having a fixed clinical preceptor during internship (*β* = −0.078, *p* = 0.009) were significantly negatively associated with burnout. In contrast, experience of medical disputes (*β* = 0.131, *p* < 0.001) was significantly positively associated with burnout. Additionally, medical students from higher-level institutions reported greater burnout (*β* = 0.144, *p* < 0.001). Sex, age, specialty type, household income level, and relationship status exhibited no statistically significant associations with the total burnout score. Multicollinearity diagnostics showed that the variance inflation factors (VIFs) of all independent variables ranged from 1.020 to 1.252, all less than 2. Detailed results are presented in Table 4. Correlation analyses for the three subdimensions (emotional exhaustion, depersonalization, and reduced personal accomplishment) are presented in Supplementary Tables S2–S4.

**Table 4:** Multiple linear regression analysis of factors associated with burnout.

Total burnout score	*B*	SE	*β*	*t*	*p*	VIF
Constant	78.867	7.992		9.869	0	
Sex	0.066	1.226	0.002	0.053	0.957	1.058
Age	−0.525	0.364	−0.046	−1.444	0.149	1.189
Specialty	−0.062	0.2	−0.009	−0.310	0.757	1.085
Institutional level	3.847	0.867	0.144	4.435	0	1.252
Scholarship experience	−2.364	1.140	−0.062	−2.074	0.038	1.045
Having a fixed clinical preceptor during the internship	−2.967	1.139	−0.078	−2.605	0.009	1.071
Having clearly defined career goals	−9.857	1.227	−0.241	−8.031	0	1.069
Experience of medical disputes	8.612	1.932	0.131	4.459	0	1.020
Household income range	−0.294	0.645	−0.013	−0.455	0.649	1.020
Relationship status	0.684	1.217	0.017	0.562	0.574	1.034

*B*, unstandardized regression coefficient; SE, standard error; *β*, standardized regression coefficient; *t*, *t* statistic; VIF, variance inflation factor. The total burnout score was entered as the dependent variable. All tests were two-sided, and *p* < 0.05 was considered statistically significant.

### 3.3 Qualitative interview findings on burnout

The interview data were organized and analyzed according to the three dimensions of the burnout scale. The findings showed that medical interns’ experiences of burnout primarily involved emotional exhaustion, depersonalization, and reduced personal accomplishment. The interview data illustrated the specific manifestations of burnout experienced by medical interns during clinical training, showed as Table 5.

**Table 5:** Manifestations of burnout experienced by medical interns during clinical training.

Category	Illustrative quotes
1. Emotional fatigue	
Physical exhaustion	“After work, I am very physically and mentally tired. Female_P9” “My body is very tired; I feel more physically and mentally tired. Male_P4”
Mental fatigue	“Repetitive, mechanical work. Pretty tired. Male_P15” “It’s tiring to be around people. Female_P11” “You do not want to read more, but you have to finish it. Male_P13” “He probably does not want me to study in it. I’m a little disappointed in that department. Male_P18”
2. Work-Related Apathy	“If I have free time, I will go to watch, on the child crying there. And I’d watch. Female_P11” “No contact with the patient…he has only one name. Female_P11” “Most of the time is boring, occasionally a bit of fresh and interesting. I do not know anything about patients. Female_P21”
3. Lack of job satisfaction	
Lacking empathy	“I do not understand why people like that exist. Male_P18”
Lack of a sense of gain	“He trusts Baidu but not the doctor. Female_P9” “Some work feels like repetitive work, but it does not mean much. Male_P15” “Out-patient pharmacies really do not seem very useful for themselves. Female_P1” “It’s all manual labor. You do not need a brain to. Just get someone to come over and do the work. Male_P18”
No emotional value	“I do not like it, I do not want to work in a hospital. I do not want to be in this patient’s environment. Male_P13” “I’m tired to death from applying the medicine. Let me take an inventory. I’m exhausted. Female_P17” “Yeah, I’m pretty tired. Monday to Saturday, Saturday, too. Male_P15”

#### 3.3.1 Emotional exhaustion: coexisting physical fatigue and mental depletion

The interview findings showed that emotional exhaustion was primarily manifested as physical fatigue and mental depletion. Some interns described it as physical tiredness and psychological exhaustion following clinical training. Several participants reported that prolonged clinical work, continuously remaining on duty, and repetitive tasks often left them feeling “physically and mentally exhausted” after work. Other participants indicated that they were required to spend long periods in clinical departments during their internships. The relatively fixed work schedule reduced their personal and rest time, resulting in pronounced feelings of fatigue and pressure. In addition to physical fatigue, the interns reported varying degrees of mental depletion. Some participants stated that, when faced with continuous clinical tasks and internship requirements, they sometimes felt that they “did not want to continue reviewing the cases” but “still had to complete the tasks”.

#### 3.3.2 Depersonalization: limited patient contact and emotional detachment

Depersonalization was primarily manifested as emotional detachment from patients, clinical tasks, and the internship environment. Some interns reported that they had limited knowledge of patients’ specific circumstances and that patients were often perceived merely as a “name” or a clinical case during the internship, rather than as individuals with complete illness experiences and emotional needs. Some participants also expressed an attitude of indifference toward clinical tasks, stating that “it did not matter” or that “it made no difference who performed the task.” The interview data showed that some interns exhibited indifference and detachment during their interactions with patients and participation in clinical tasks. Their accounts mainly reflected limited understanding of patients, restricted clinical involvement, and insufficient emotional engagement in work-related tasks.

#### 3.3.3 Reduced personal accomplishment: repetitive work undermining the perceived value of the profession

The interview findings showed that reduced personal accomplishment was primarily manifested as difficulty deriving a sufficient sense of value, growth, and professional identity from clinical training. Some interns described their clinical tasks as being like an “assembly line” or as “repetitive work,” perceiving certain tasks as mechanical and monotonous and as having little direct relevance to the development of professional competence. Other participants stated that some tasks were “entirely physical work” and did not allow them to demonstrate the value of their professional knowledge and abilities. Some participants further indicated that the tasks they completed at work “had no value” and expressed uncertainty about whether they would continue working in a hospital in the future. The relevant interview accounts mainly focused on repetitive work, an insufficient sense of work-related value, limited opportunities to apply professional competence, and inadequate experiences of professional growth.

### 3.4 Integration of quantitative and qualitative findings

Integration of the quantitative and qualitative findings for each dimension of burnout revealed that, in the quantitative phase, burnout among medical interns was mainly manifested as emotional exhaustion, depersonalization, and reduced personal accomplishment, all of which were significantly positively correlated with the total burnout score (Supplementary Table S5). On this basis, the qualitative phase provided concrete representations of these three dimensions: emotional exhaustion was primarily manifested as physical fatigue, mental depletion, prolonged duty hours, and insufficient rest; depersonalization was primarily manifested as limited understanding of patients, emotional detachment, and indifference toward clinical tasks; and reduced personal accomplishment was primarily manifested as repetitive work experiences, an insufficient sense of work-related value, and inadequate professional growth. The quantitative phase clarified the relationships among the dimensions of burnout, whereas the qualitative phase provided in-depth exploration of the content of each dimension. Overall, burnout among medical interns was not limited to a single experience of fatigue but rather represented a multidimensional phenomenon involving physical and mental exhaustion, emotional detachment, and a diminished sense of value. Emotional exhaustion, depersonalization, and reduced personal accomplishment collectively constituted the principal features of burnout among medical interns.

## 4 Discussion

To our knowledge, this is the first study in mainland China to use an explanatory sequential mixed-methods design to examine burnout among medical interns. Burnout was distinctly multidimensional, mainly involving emotional exhaustion, depersonalization, and reduced personal accomplishment, with emotional exhaustion showing the strongest correlation with the total burnout score. Institutional level, scholarship experience, having a fixed clinical preceptor, having clearly defined career goals, and experience of medical disputes were significantly associated with burnout. Qualitative interviews further indicated that prolonged working hours, repetitive tasks, limited patient contact, insufficient preceptor feedback, and a lack of perceived professional growth were important contextual contributors. These findings are broadly consistent with previous studies reporting emotional exhaustion, depersonalization, and reduced personal accomplishment as the core dimensions of burnout among medical trainees (Di Vincenzo et al., 2024; Monti et al., 2025). However, unlike studies focusing mainly on quantitative associations, the present mixed-methods design shows how these dimensions are experienced in daily clinical work: emotional exhaustion is closely tied to long duty hours and insufficient rest, whereas depersonalization may function as a protective response to sustained emotional overload.

The clinical internship is an important transitional period during which medical students move from classroom learning to clinical practice and gradually shift from student to healthcare professional (Carlsson et al., 2023). Interns must cope with patient interactions, departmental tasks, preceptor expectations, and internship assessments, while their clinical competence, professional autonomy, and decision-making authority are still developing, which may lead to role ambiguity and a mismatch between responsibilities and capabilities (Funer et al., 2024). Prolonged working hours, insufficient rest, and repetitive tasks may initially result in physical fatigue and mental depletion. When such exhaustion persists without effective support, interns may protect themselves by reducing emotional engagement, leading to detachment from patients, clinical tasks, and the internship environment (Moreira et al., 2025). When clinical training is persistently experienced as mechanical, repetitive, and lacking opportunities for professional growth, interns may find it difficult to derive a sense of value and accomplishment (Ten Cate et al., 2024). These findings suggest that burnout does not arise solely from insufficient individual coping capacity, but develops through the accumulation of physical and mental depletion, emotional detachment, and a diminished sense of value during the transition to clinical practice (Monti et al., 2025). This interpretation is supported by previous research indicating that role ambiguity and the gap between responsibility and competence are important drivers of burnout in clinical training settings (Cho et al., 2024; Carlsson et al., 2023).

Among the associated factors, having clearly defined career goals showed the strongest association with burnout, suggesting that clarity of goals and a perceived pathway for professional development may shape the internship experience (Chaillet et al., 2025; Dioquino et al., 2024). This is broadly in line with previous research showing that career clarity and perceived professional development are protective against burnout (Dioquino et al., 2024). One possible explanation is that interns with clear career goals are more likely to interpret clinical tasks as meaningful steps toward their professional identity, whereas those without clear goals may experience clinical work as passive and repetitive. A fixed clinical preceptor may influence interns’ engagement by providing consistent guidance, timely feedback, and a professional role model (Amaral and Figueiredo, 2023). Experience of medical disputes reflects the influence of adverse clinical events on professional socialization and may increase concerns regarding future occupational risks and clinician–patient relationships. Scholarship experience and specialty classification further suggest that previous academic performance, professional identity, and differences in clinical participation across specialties may influence how interns adapt to clinical work (Hora et al., 2024). By contrast, some studies have reported that demographic and academic factors, such as gender or academic performance, play a more prominent role (Priyam et al., 2024). These differences may reflect variations in healthcare systems, internship management models, and cultural contexts.

The present findings also have direct implications for the succeeding phase of medical training. Burnout is not confined to the internship: observational studies report a substantial prevalence of burnout among resident doctors, with one single-center study reporting that 42.4% of residents met criteria for burnout (Shahi et al., 2022), and another single-center study reporting that 75% of postgraduate residents experienced burnout (Priyam et al., 2024). Reviews of residency training have repeatedly identified time and workload demands, limited control over one’s work, and the quality of interpersonal and supervisory relationships as central contributors to burnout (Ishak et al., 2009). Several of these drivers, heavy workload, limited autonomy, and the nature of preceptor and supervisory relationships, overlap with the contextual factors associated with burnout in our intern sample, suggesting that the mechanisms underlying intern burnout are likely to persist and intensify as trainees assume greater clinical responsibility. This is consistent with the observation that the formative years of training are a period of heightened vulnerability during which early recognition and intervention are particularly valuable (Priyam and Sil, 2020). At the same time, a recent commentary accompanying a national study of resident work hours cautions that the relationship between workload and burnout is not straightforward, and that a balance between clinical demands and wellbeing must be actively managed during residency (Tillou and Hiatt, 2026). Taken together, these considerations support our proposal that burnout should be addressed as a continuum spanning the student-to-doctor transition, through coordinated, staged interventions—career-planning guidance, optimized preceptor support, and structured support after adverse events—that begin during the internship and extend into residency, rather than through measures confined to either phase in isolation (Ishak et al., 2009; Priyam and Sil, 2020).

These findings suggest that interventions should not be limited to individual psychological adjustment but should also focus on internship management and the medical education support system (Zhang et al., 2026). First, internship administrators should help students clarify their stage-specific learning objectives, role responsibilities, and career development directions before the internship and during clinical rotations (Zou et al., 2024), and internship tasks should be aligned with competency-development objectives to reduce interns’ feelings of passivity and meaninglessness toward clinical work. Second, the fixed-preceptor system should be further optimized. Attention should be given not only to whether a fixed preceptor is assigned but also to the quality of supervision, the frequency of feedback, and the effectiveness of interactions between preceptors and interns, thereby strengthening interns’ sense of support and professional growth during clinical practice. Third, low-value, mechanical, and repetitive tasks should be appropriately reduced, while interns should be provided with more opportunities to participate in case discussions, patient communication, clinical observation, and professional reflection (Zhang et al., 2026). Such opportunities may enable them to develop professional competence and a sense of occupational value in authentic clinical settings. Finally, for interns who have experienced medical disputes, conflicts with patients, or marked emotional exhaustion, mechanisms should be established for the timely review of adverse events, psychological support, and communication-skills training (Lindig et al., 2024). These measures may help interns transform negative clinical experiences into learning opportunities rather than allowing such experiences to develop into occupational fear and emotional detachment.

This study has several limitations. First, it was conducted at a large tertiary hospital in central China, and all participants were recruited from the same internship management system. Therefore, caution is warranted when generalizing the findings to other regions, hospitals at different levels, or students from different medical education backgrounds. Another limitation is that we did not apply formal exclusion criteria for anxiety or depressive symptoms or disorders, or for other medical conditions. These unmeasured psychological and physical health conditions may act as confounding variables and influence burnout outcomes. Second, the quantitative component used a cross-sectional design, which could identify associations between relevant factors and burnout but could not establish causal relationships (Ryan et al., 2023). Third, burnout and its associated factors were assessed primarily through interns’ self-reports and may therefore have been subject to recall bias, social desirability bias, and common method bias. Fourth, the qualitative component prioritized interviews with interns who had relatively high levels of burnout, and selecting interview participants using a composite score threshold for qualitative part of this study may have influenced the range of qualitative experiences gathered. Although this approach facilitated an in-depth exploration of burnout experiences, it may have provided insufficient insight into the positive adaptation experiences of interns with low levels of burnout. Future studies should adopt multicenter and longitudinal designs and incorporate assessments of the clinical learning environment, evaluations by clinical preceptors, and internship performance data to further examine the mechanisms underlying burnout among medical interns and to explore interventions targeting career planning, preceptor support, and adverse clinical experiences.

## Data Availability

The raw data supporting the conclusions of this article will be made available by the authors, without undue reservation.
