# Supplementary material for: Job burnout among medical interns: a mixed-methods study of current status, associated factors, and experiential characteristics
Source: Front Psychol. 2026 Oct 7;17:1922425. doi: 10.3389/fpsyg.2026.1922425 (PMC13683102; doi:10.3389/fpsyg.2026.1922425)
Supplement: Supplementary file 1 [file Table_1.DOCX]

**Table S1 Consolidated criteria for reporting qualitative studies (COREQ): 32-item checklist**

| No Item | Guide questions/description | Answers |
| --- | --- | --- |
| **Domain 1: Research team and reflexivity** | | |
| Personal Characteristics | | |
| 1. Interviewer/facilitator | Which author/s conducted the interview or focus group? | Zheng Li, Yifan Song, Xiaojing Lu and Zhiyong Sun |
| 2. Credentials | What were the researcher’s credentials? E.g. PhD, MD | Zheng Li, MS; Yifan Song, MS; Xiaojing Lu, MS; Zhiyong Sun, MS |
| 3. Occupation | What was their occupation at the time of the study? | Hospital pharmacists |
| 4. Gender | Was the researcher male or female? | Zheng Li, Male; Yifan Song, Male; Xiaojing Lu, Female; Zhiyong Sun, Male |
| 5. Experience and training | What experience or training did the researcher have? | Theoretical training and experience of conducting several qualitative studies with other groups. |
| Relationship with participants | | |
| 6. Relationship established | Was a relationship established prior to study commencement? | Yes |
| 7. Participant knowledge of the interviewer | What did the participants know about the researcher? e.g. personal goals, reasons for doing the research | Reasons for doing the research |
| 8. Interviewer characteristics | What characteristics were reported about the interviewer/facilitator? e.g. Bias, assumptions, reasons and interests in the research topic | Reasons and interests in the research topic |
| **Domain 2: study design** | | |
| Theoretical framework | | |
| 9. Methodological orientation and Theory | What methodological orientation was stated to underpin the study? e.g. grounded theory, discourse analysis, ethnography, phenomenology, content analysis | Mixed methods (quantitative and qualitative). The qualitative component used thematic analysis.. |
| Participant selection | | |
| 10. Sampling | How were participants selected? e.g. purposive, convenience, consecutive, snowball | Purposive |
| 11. Method of approach | How were participants approached? e.g. face-to-face, telephone, mail, email | face-to-face or telephone |
| 12. Sample size | How many participants were in the study? | 23 |
| 13. Non-participation | How many people refused to participate or dropped out? Reasons? | a total of 25 eligible trainees were approached, of whom 23 participated (2 declined) |
| Setting | | |
| 14. Setting of data collection | Where was the data collected? e.g. home, clinic, workplace | face-to-face |
| 15. Presence of non-participants | Was anyone else present besides the participants and researchers? | No |
| 16. Description of sample | What are the important characteristics of the sample? e.g. demographic data, date | Yes, demographic data. |
| Data collection | | |
| 17. Interview guide | Were questions, prompts, guides provided by the authors? Was it pilot tested? | Yes |
| 18. Repeat interviews | Were repeat interviews carried out? If yes, how many? | No |
| 19. Audio/visual | Did the research use audio or visual recording to collect the data? | Audio recording |
| 20. Field notes | Were field notes made during and/or after the interview or focus group? | Yes |
| 21. Duration | What was the duration of the interviews or focus group? | Approximately 40 minutes. |
| 22. Data saturation | Was data saturation discussed? | Yes |
| 23. Transcripts returned | Were transcripts returned to participants for comment and/or correction? | Yes |
| **Domain 3: analysis and findingsz** | | |
| Data analysis | | |
| 24. Number of data coders | How many data coders coded the data? | Two |
| 25. Description of the coding tree | Did authors provide a description of the coding tree? | Yes |
| 26. Derivation of themes | Were themes identified in advance or derived from the data? | Derived from the data |
| 27. Software | What software, if applicable, was used to manage the data? | NVIVO 12 |
| 28. Participant checking | Did participants provide feedback on the findings? | Yes |
| Reporting | | |
| 29. Quotations presented | Were participant quotations presented to illustrate the themes / findings? Was each quotation identified? e.g. participant number | Yes |
| 30. Data and findings consistent | Was there consistency between the data presented and the findings? | Yes |
| 31. Clarity of major themes | Were major themes clearly presented in the findings? | Yes |
| 32. Clarity of minor themes | Is there a description of diverse cases or discussion of minor themes? | Yes |

**Table S2. Multiple Linear Regression Analysis of Factors Associated With Emotional Exhaustion**

| **Emotional Exhaustion** | **B** | **SE** | **β** | **t** | **P** | **VIF** |
| --- | --- | --- | --- | --- | --- | --- |
| Constant | 27.859 | 4.149 |  | 6.715 | 0 |  |
| Sex | -0.786 | 0.636 | -0.037 | -1.236 | 0.217 | 1.058 |
| Age | -0.335 | 0.189 | -0.057 | -1.775 | 0.076 | 1.189 |
| Specialty | -0.01 | 0.104 | -0.003 | -0.094 | 0.925 | 1.085 |
| Classification of the specialty within the university | 2.682 | 0.45 | 0.197 | 5.956 | 0 | 1.252 |
| Scholarship experience | -0.633 | 0.592 | -0.032 | -1.07 | 0.285 | 1.045 |
| Having a fixed clinical preceptor during the internship | -1.03 | 0.591 | -0.053 | -1.741 | 0.082 | 1.071 |
| Having clearly defined career goals | -2.948 | 0.637 | -0.141 | -4.626 | 0 | 1.069 |
| Experience of medical disputes | 4.587 | 1.003 | 0.136 | 4.575 | 0 | 1.02 |
| Household income range | -0.411 | 0.335 | -0.037 | -1.228 | 0.22 | 1.02 |
| Relationship status | 0.742 | 0.632 | 0.035 | 1.175 | 0.24 | 1.034 |

**Table S3. Multiple Linear Regression Analysis of Factors Associated With Depersonalization**

| **Depersonalization** | **B** | **SE** | **β** | **t** | **P** | **VIF** |
| --- | --- | --- | --- | --- | --- | --- |
| Constant | 12.617 | 2.485 |  | 5.078 | 0 |  |
| Sex | -1.256 | 0.381 | -1 | -3.296 | 0.001 | 1.058 |
| Age | -1.22 | 0.113 | -0.035 | -1.082 | 0.279 | 1.189 |
| Specialty | -.061 | 0.062 | -0.03 | -0.977 | 0.329 | 1.085 |
| Classification of the specialty within the university | 1.069 | 0.270 | 0.131 | 3.964 | 0 | 1.252 |
| Scholarship experience | -1.007 | 0.354 | -0.086 | -2.843 | 0.005 | 1.045 |
| Having a fixed clinical preceptor during the internship | -0.747 | 0.354 | -0.065 | -2.11 | 0.035 | 1.071 |
| Having clearly defined career goals | -1.706 | 0.382 | -0.137 | -4.47 | 0 | 1.069 |
| Experience of medical disputes | 2.319 | 0.601 | 0.115 | 3.862 | 0 | 1.02 |
| Household income range | 0.174 | 0.201 | 0.026 | 0.865 | 0.387 | 1.02 |
| Relationship status | 0.102 | 0.378 | 0.008 | 0.268 | 0.799 | 1.034 |

**Table S4. Multiple Linear Regression Analysis of Factors Associated With Reduced Personal Accomplishment**

| **Reduced Personal Accomplishment** | **B** | **SE** | **β** | **t** | **P** | **VIF** |
| --- | --- | --- | --- | --- | --- | --- |
| Constant | 38.391 | 3.805 |  | 10.089 | 0 |  |
| Sex | 2.108 | 0.584 | 0.109 | 3.611 | 0 | 1.058 |
| Age | -0.068 | 0.173 | -0.012 | -0.39 | 0.697 | 1.189 |
| Specialty | 0.009 | 0.095 | 0.003 | 0.09 | 0.929 | 1.085 |
| Classification of the specialty within the university | 0.096 | 0.413 | 0.008 | 0.233 | 0.816 | 1.252 |
| Scholarship experience | -0.724 | 0.543 | -0.04 | -1.334 | 0.183 | 1.045 |
| Having a fixed clinical preceptor during the internship | -1.191 | 0.542 | -0.067 | -2.195 | 0.028 | 1.071 |
| Having clearly defined career goals | -5.204 | 0.584 | -0.271 | -8.903 | 0 | 1.069 |
| Experience of medical disputes | 1.705 | 0.92 | 0.055 | 1.854 | 0.064 | 1.02 |
| Household income range | -0.056 | 0.307 | -0.005 | -0.182 | 0.856 | 1.02 |
| Relationship status | -0.16 | 0.579 | -0.008 | -0.275 | 0.783 | 1.034 |

**Table S5. Integration of the quantitative and qualitative findings for the different dimensions of burnout.**

| **MBI Domains** | **Ouantitative findings, r (P)** | **Qualitative findings** | **Meta-inferences** |
| --- | --- | --- | --- |
| Emotional exhaustion | Significantly positively correlated with the total burnout score, showing the strongest correlation among the three dimensions.  0.849 (<0.01) | Physical fatigue, mental depletion, continuous on-duty work, and insufficient rest. | The strongest correlation between emotional exhaustion and the total burnout score is reflected in medical interns’ physical fatigue, mental depletion, continuous on-duty work, and insufficient rest. |
| Depersonalization | Significantly positively correlated with the total burnout score, with a strong correlation.  0.803 (<0.01) | Limited understanding of patients, emotional detachment, and indifferent attitudes toward clinical tasks. | The strong correlation between depersonalization and the total burnout score is reflected in medical interns’ limited understanding of patients, emotional detachment, and indifferent attitudes toward clinical tasks. |
| Reduced personal accomplishment | Significantly positively correlated with the total burnout score.  0.723 (<0.01) | Repetitive work, insufficient sense of work-related value, and inadequate professional growth. | The significant positive correlation between reduced personal accomplishment and the total burnout score is reflected in medical interns’ experience of repetitive work, insufficient sense of work-related value, and inadequate professional growth. |
| Overall integrated findings | All three dimensions (emotional exhaustion, depersonalization, and reduced personal accomplishment) were significantly positively correlated with the total burnout score.  (r = 0.682~0.849, all P < 0.01) | Burnout among medical interns is not merely a feeling of fatigue, but a multidimensional phenomenon involving physical and mental depletion, emotional detachment, and diminished sense of value. | The significant positive correlations between the three dimensions and the total burnout score collectively indicate that burnout among medical interns is not merely a feeling of fatigue, but a multidimensional phenomenon characterized by physical and mental depletion, emotional detachment, and diminished sense of value. |
